# Synthesis of cellulose nanofibers from jute fiber by using chemomechanical method

**DOI:** 10.12688/f1000research.138665.1

**Published:** 2024-01-08

**Authors:** Siti Syazwani N., Ervina Efzan M.N., Kok C.K., Nurhidayatullaili M.J.

**Affiliations:** 1 Faculty of Engineering and Technology (FET), Multimedia University, Malacca, Malacca, 75050, Malaysia; 2 Nanotechnology Catalyst and Research Centre (NanoCAT), Universiti Malaya, Kuala Lumpur, Federal Territory of Kuala Lumpur, 50603, Malaysia

**Keywords:** fiber, cellulose, nanomaterials, synthesised, agro-waste, jute

## Abstract

**Background:**

Jute fiber is one of the most versatile natural fibers that is widely used as a raw material for packaging, textiles, and construction; and as a reinforcement in composite materials for heavy-duty applications. In the past, acid hydrolysis and mechanical treatment via the ball milling method were common in the extraction of cellulose nanofiber (CNFs) from natural plant fibers. However, there are some drawbacks of using those methods where there will be a huge quantity of acidic wastewater generated when the acid hydrolysis method is performed.

**Method:**

This study investigated the potential use of a combination of chemical and mechanical methods in the extraction of jute CNFs. Through this method, the jute fibers were first chemically treated using sodium hydroxide (NaOH), sodium chlorite (NaClO _2_) and sulphuric acid (H _2_SO _4_) to remove the non-cellulosic elements followed by mechanical milling by using a planetary ball mill.

**Results:**

The shape and size of the obtained CNFs were observed under a field emission scanning electron microscope (FESEM). This study revealed that jute CNFs were successfully extracted through the combination of chemical and mechanical treatment methods where the obtained CNFs reveal themselves in smooth fibrous morphology with a diameter of 23 nm and 150-200nm in length.

**Conclusions:**

Jute cellulose nanofibers were successfully drawn out from raw jute fibers by means of a combination of chemical and mechanical treatment. The results obtained confirmed that the chemomechanical method is an effective technique for isolating the CNFs and its potential use as reinforcement material was explained.

## Introduction

The demand for environmentally friendly products and processes in various fields such as electronic devices, automotive, building construction, military defences and aircraft is increasing. Thus, the exploitation of agro-industry by-products such as rice husk, banana peels, hemp, jute and bamboo in developing new materials is expanding. Cellulose and fiber are considered the most abundant bio-based polymers in nature. Cellulose is the core constituent of all plant fibers and has repeated links to β-D-glucopyranose ( Nagarajan *et al.*, 2019). The utilization of nanocellulose as reinforcing materials in polymer matrices has gained wide attention in the material research community due to their availability, low cost and ease of extraction processing. Recent literature ( Wang *et al.*, 2017; Meng *et al.*, 2019; Harini *et al.*, 2018) suggests that nanocellulose has superior properties such as high interfacial area and strength, large surface-to-volume ratio, low density, high tensile strength, high stiffness and flexibility; good dynamic mechanical and thermal properties which make them suitable as reinforcing materials in polymeric materials to grant excellent improvement in properties of the fabricated polymer nanocomposites.

Nanoscale structural cellulose can be illustrious into three types namely cellulose nanocrystal (CNC), cellulose nanofiber (CNF) and bacterial cellulose (BC). However, the most common types that are widely used in engineering are CNC and CNF. The main distinctions between these two types of nanocellulose are their dimensions ( Meng *et al.*, 2019) and crystallinity. CNF contains both amorphous and crystalline cellulose up to few micrometers in length. They have a larger surface area with a net-like or web-like structure than CNC, which makes them a promising reinforcing agent for polymer composites. On the other hand, CNC contains 100% cellulose content resulting from the absence of amorphous regions and has elongated, crystalline, rod-like shapes with limited flexibility compared to CNF.

Apart from properties, the other typical difference between CNF and CNC is in their extraction methods. Many studies ( Meng *et al.*, 2019; Nagarajan *et al.*, 2019; Phanthong *et al.*, 2018; Dahlem *et al.*, 2019) show that the different techniques of extraction from respective sources will result in the production of cellulose nanoparticles with varying crystal structures, morphologies, surface interactions and other specific properties. Usually, CNC is extracted from agro-waste by using chemical means such as acid hydrolysis ( Danial *et al.*, 2015), alkaline treatment ( Melikoğlu *et al.*, 2019), microwave-assisted step ( Singh *et al.*, 2017) and enzymatic hydrolysis ( Paulo *et al.*, 2019). Acid hydrolysis is the most used method in extracting CNC from cellulose fiber. It is usually initiated by alkaline treatment and bleaching before acid treatment. Several types of acid are widely employed in acid hydrolysis methods, such as sulfuric acid, hydrochloric acid, phosphoric acid and hydrobromic acid. However, the most frequently used acid is hydrochloric acid due to its ability to strongly isolate the nanocrystalline cellulose and make it dispersed as a stable colloid system ( Phanthong *et al.*, 2018; Lu and Hsieh, 2010; Das *et al.*, 2009).

Meanwhile, the extraction method of CNF can be categorized into three types, namely mechanical treatments (homogenization, grinding and ball milling); a combination of biological and chemical pretreatments (enzymatic hydrolysis and TEMPO-mediated oxidation); and the combination of mechanical and chemical treatments (chemomechanical treatment). Usually, CNF is extracted from agro-waste materials with the primary aim to turn waste into excellent materials and directly save the environment. Thus, the energy impact that comes with the implementation of the mechanical method alone is not preferable. Instead, the combined use of mechanical and chemical treatment can reduce the amount of energy used in mechanical processes, making the production of CNF more economically viable ( Dahlem *et al.*, 2019; Mishra *et al.*, 2018).

CNFs have been widely extracted from plant fibers such as banana peel and bract ( Harini *et al.*, 2018; Meng *et al.*, 2019), pineapple leaf ( Ravindran *et al.*, 2019), kenaf bast ( Karimi *et al.*, 2014; Kian *et al.*, 2018) and cotton ( Maciel *et al.*, 2018; Morais *et al.*, 2013) by using chemical approaches (acid hydrolysis method) which lead to disposal of acidic wastewater produced during the washing process to neutralize the pH of the suspension and also incur high cost. Hence, to reduce energy consumption and chemical usage, this work was done to obtain CNF from jute fiber using a chemomechanical method. Chemical treatment such as alkaline treatment, bleaching and acid treatment was involved in the extraction process followed by mechanical treatment using a milling process. Table 1 shows the lignocellulosic content of jute and some other fibers.

**Table 1.** Lignocellulosic content of jute and some other fibers.

Fibers	Cellulose (wt%)	Hemicellulose (wt%)	Pectin (wt%)	Lignin (wt%)	Wax (wt%)	Reference
Jute	73.5	20.1	1.8	3.1	0.5	Tanmoy *et al.*, 2014
Flax	66	15	5	4	1.7	Aslan and Mustafa, 2012
Hemp	44.5	32.78	3.57	21.03	1.3	Stevulova *et al.*, 2014
Wood	49	20	-	29	-	Aslan, 2012
Cotton	81	13	0.9	1.3	0.6	Lewin, 2007

Based on Table 1, it is shown that jute has a low content of lignin, pectin and waxes compared to other types of fibers, which suggests it can easily undergo a bleaching process and have a high fiber strength.

## Methods

Jute is a type of bast fiber that was drawn out from corchorus plants. It is produced in large quantities and is known as one of the cheapest natural fibers. In this work, a natural jute rope hemp was used as the main material. The natural jute fiber used was received from Zhejiang Hailun Rope and Net Co., LTD. While the chemicals used in the extraction process such as H _2_SO _4_ and NaClO _2_ were received in liquid form from Merck (1007131000 and 8148151000). On the other hand, the NaOH was received in pellet form from Chemiz.

The jute fibers were cut into pieces 2 to 3 cm long before the swelling process. During the swelling process, 25 g of jute fibers were immersed in 250 ml of 18% sodium hydroxide (NaOH) solution for 2 hours to attenuate their structure. The swollen fibers were then filtered and washed with an excess amount of distilled water and neutralized, followed by a drying process at room temperature. The swollen fibers were then hydrolyzed in 250 ml of 2M sulphuric acid (H _2_SO _4_) at 80°C for 3 hours with continuous stirring. Then, they were filtered and rinsed with distilled water after stirring in the 250 ml H _2_SO _4_ for 3 hours until the pH became neutral. The hydrolyzed fiber was then subjected to alkaline treatment to eliminate the soluble lignin in the fibers by treating them with NaOH at 80°C for 2 hours with continuous stirring and rinsing with distilled water before air drying for 3 hours. The dried fibers were bleached with 250 ml of 2% sodium chlorite solution (NaClO _2_) at 50°C for 1 hour to eliminate the remaining lignin. This treatment was repeated 2 to 3 times until the residue became colorless. Distilled water was used to cool, rinse and filter the reaction mixture to neutral before drying in air.

Then, the mechanical process took place in the extraction process, in which the extracted fibers from chemical treatment were milled. The grinding process was carried out in the wet state using a Retsch-PM 100 high-energy planetary mill. A wet grinding process was conducted in deionized water using a 10 mm diameter zirconia ball. The vessel was filled with a ball-to-material ratio of 10:1 and deionized water and ground at room temperature with a speed of 450 rpm for 6 hours. After grinding, the slurry was collected for the drying process. The slurry was then dried using a freeze dryer at -50°C for 8 hours. A field emission scanning electron microscope (FESEM) was used to analyze the size and morphology of the extracted Jute CNFs at 500, 1 and 20k magnification.

## Results and discussion

### Morphology of raw jute fibers and extracted jute CNF

There are a few methods that can be used to study the properties of nanomaterials including chemical and structure analysis (FTIR and XRD), thermal analysis (DSC and TGA) and morphology and size analysis (AFM, FESEM and TEM). However, it is found that the methods such as AFM, FESEM and TEM are effective methods to analyze the changes in the morphology and size of extracted CNF after each treatment phase (chemical and mechanical treatment) ( Ravindran *et al.*, 2019; Xie *et al.*, 2016; Tuerxun *et al.*, 2019; Pradhan and Jain, 2017). Thus, in this work, the morphology shape and size of extracted jute CNF were analyzed by using FESEM.

Microstructural analysis is one of the most vital parts in analyzing a material. This is because the shape and morphology structure of a material have tremendous effects on the physical, mechanical and thermal properties of the final material. The microstructure of raw jute fiber and extracted jute cellulose nanofiber (CNF) was analyzed by using a field emission scanning electron microscope (FESEM) at low and high magnification.

**Figure 1.**
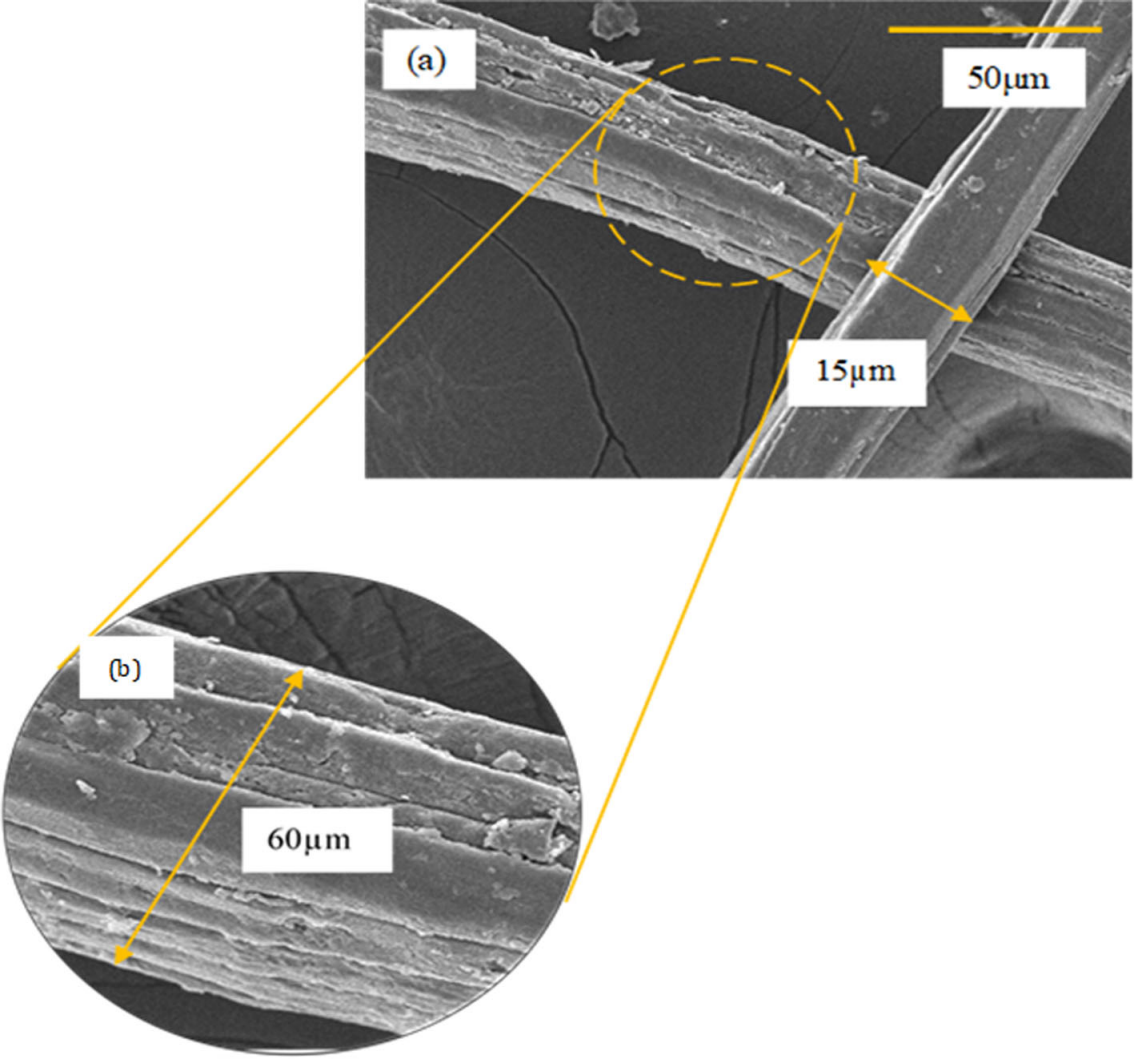
FESEM micrograph of (a) raw jute fiber at 500 magnification (b) raw jute fiber at 1k magnification.

Figure 1 (a) and (b) shows the micrograph of raw jute fiber observed under the field emission scanning electron microscope (FESEM) at low and high magnification. It was observed that the raw jute fibers present in long rod shapes with surface roughness and irregularities ( Figure 1 (a) and (b)) with dimensions ranging from 10 to 60 μm. Additionally, the rough surface and long coiled fiber bundles are visible under high magnification morphology ( Figure 1 (b)). Each elemental fiber is present in a compact structure that exhibits an orientation in the fiber axis direction with some non-fibrous elements on the surface (rough surface). The rough surface of raw jute fibers might be attributed to the existence of non-fibrous elements like waxes, lignin, hemicellulose and non-cellulosic contents. These non-cellulosic elements are known to bind the surface of cellulosic fibers and act as ‘natural binders’. Similar observations were made by Ng *et al.* (2015) and Meng *et al.* (2019) on the surface of their plant fibers.

**Figure 2.**
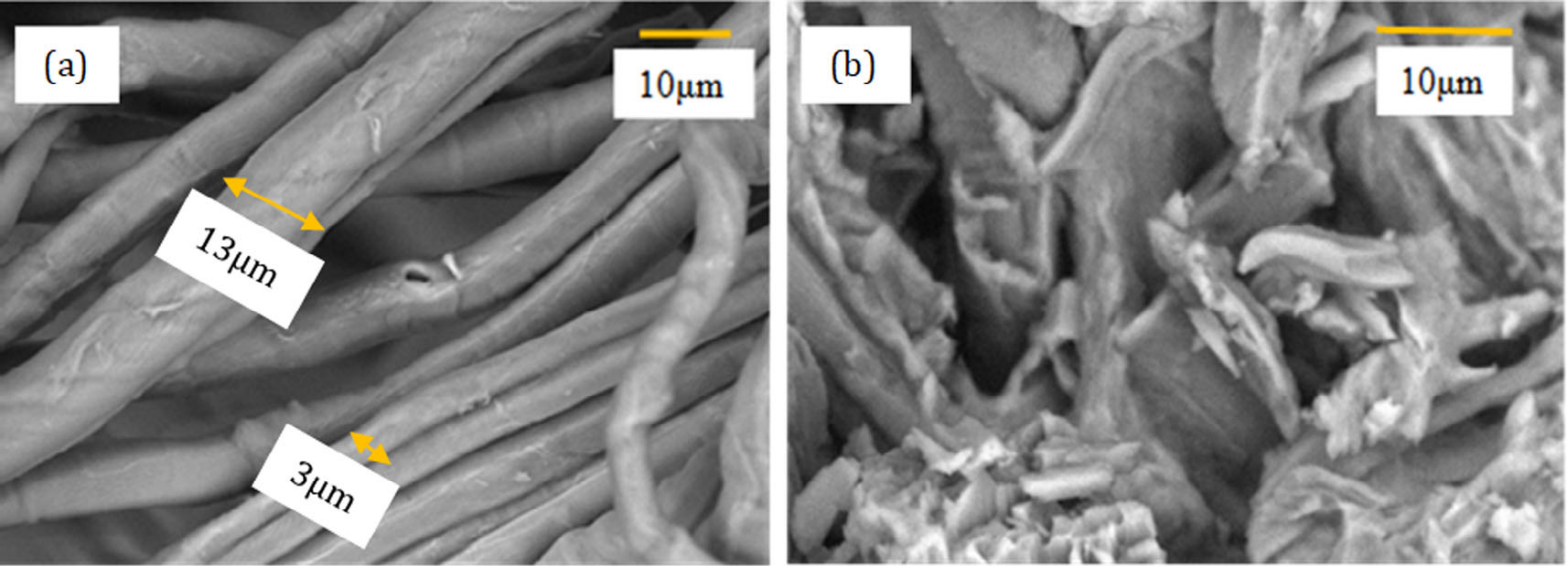
FESEM micrograph of (a) and (b) chemically treated jute fiber at 1k magnification.

Meanwhile, Figure 2 (a) and (b) show the FESEM micrograph of the jute fiber at 1k magnification after chemical treatment. It is clearly shown that the surface of chemically treated jute fibers seemed smooth and clean ( Figure 2 (a)), representing that non-cellulosic components were successfully detached. During the swelling and alkaline process, some of the individual microfibers were exposed and the fiber bundles start to loosen due to the incomplete detachment of the non-cellulosic impurities. It was noted that there were small pieces and granules on the surface of the fibers, as shown in Figure 2 (b). This might be due to the lignin polycondensate and deposits of inorganic materials during the swelling and alkaline process. However, after the bleaching process, the fibers exhibited a relatively shiny fibrous texture and a smooth surface with average diameters ranging from 3 to 13 μm.

**Figure 3.**
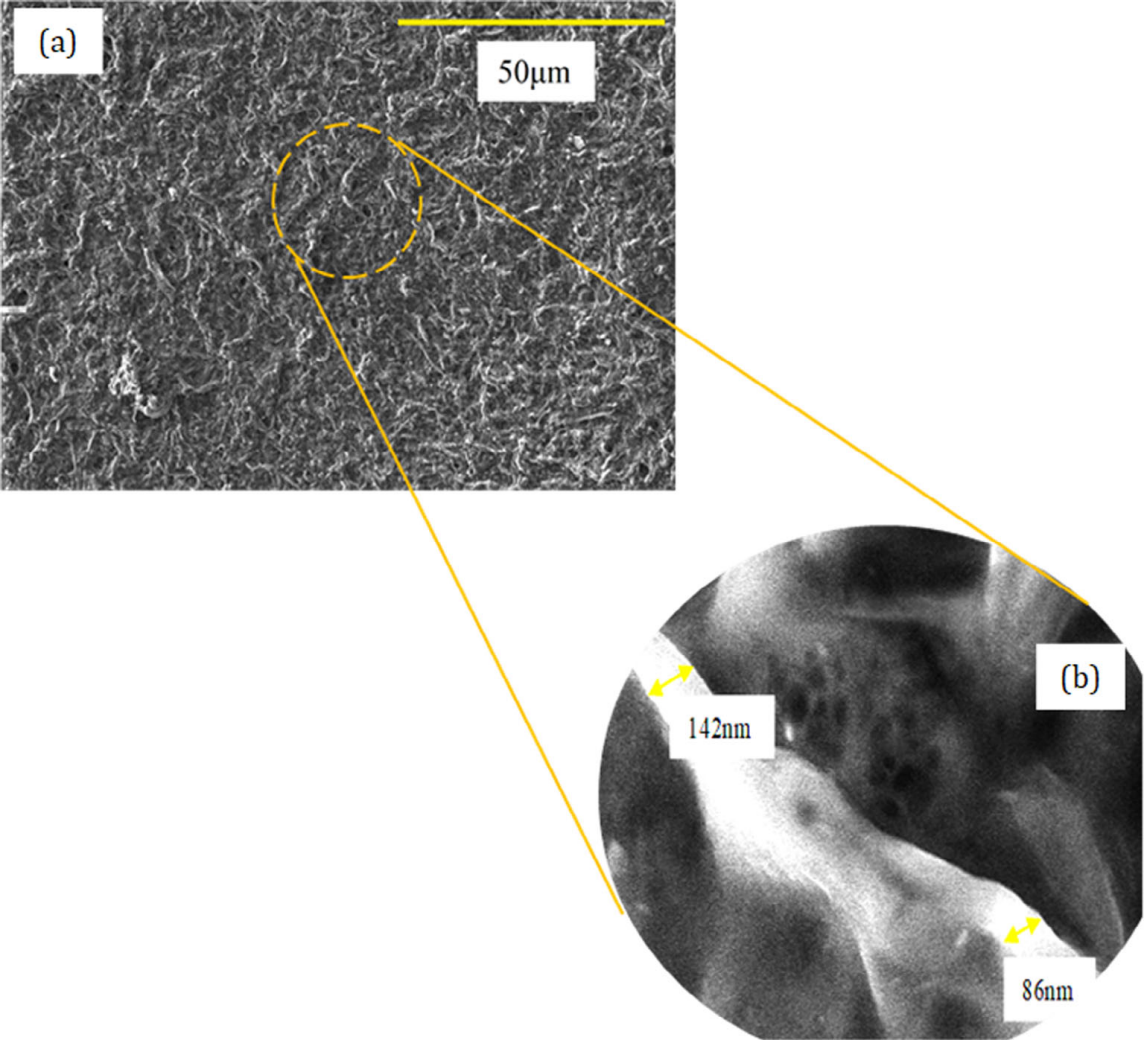
FESEM micrograph of (a) chemical and mechanically treated jute CNF at 1k magnification and (b) chemical and mechanically treated jute fiber at 20k magnification.

The extracted jute CNFs ( Figure 3 (a) and (b)) range from 23 to 300 nm in diameter at the nanoscale and have a network-like intersecting fiber morphology. These CNFs were successfully obtained via the chemomechanical method, in which the jute fibers were treated via a chemical treatment that involves swelling, alkaline treatment and bleaching process before undergoing a mechanical milling process using a planetary ball mill. The removal of non-cellulosic components from the raw jute fiber shows that the chemical treatment is successful in breaking the hydrogen bond between cellulose fibers. Furthermore, the mechanical milling process combined with the chemical treatment of jute fiber allows the removal of wax, lignin and other non-cellulosic contents thus increase in cellulose content. Therefore, this procedure is expected to play an excellent role in improving the adhesive properties of the extracted CNF in the polymer matrix. In addition, the reinforcement materials with fibrous morphology are preferred over those with spherical shapes as this morphology offers better resistance to cavity growth in the composite materials.

## Conclusion

The jute cellulose nanofibers (CNF) were successfully drawn out from raw jute fibers by means of a combination of chemical and mechanical treatment. Based on the FESEM result, it is obvious that the chemomechanical method (combination of chemical treatments and planetary ball milling) is an effective technique for isolating CNF from jute fiber. FESEM micrographs show that the extracted CNF possess itself in the nano-sized dimension of 23 to 300 nm with a network of fibrous structure crossing each other. The surface morphology of the jute fiber ( Figure 1c) is much smoother indicating that the non-cellulosic components were effectively detached via chemical treatment. Besides, the morphology of the extracted jute CNFs also shows their suitability to be used as reinforcement materials as the network fibrous shapes can offer better resistance to cavity growth and hence, improve the properties of composite materials.

## Data Availability

figshare: Synthesis of Cellulose Nanofiber from Jute Fiber via Chemomechanical Method. https://doi.org/10.6084/m9.figshare.22114919.v2 Data are available under the terms of the Creative Commons Attribution 4.0 International license (CC-BY 4.0).
