# Supplementary material for: Synthesis of cellulose nanofibers from jute fiber by using chemomechanical method
Source: F1000Res. 2024 Jan 8;13:40. [Version 1] doi: 10.12688/f1000research.138665.1 (PMC11380075; doi:10.12688/f1000research.138665.1)
Supplement: Peer review history

# Reviewer report: Reviewer response for version 1

**DOI:** 10.5256/f1000research.151883.r308126

**Published:** 2024-09-06

**Authors:** Dr. Ashok Kumar Bharimalla

**Affiliations:** 1 ICAR-Central Institute for Research on Cotton Technology, Mumbai, India

The manuscript may be accepted after a major revision:

 1) The FTIR and XRD characterization of cellulose extracted from the jute fiber is very much essential to check the quality/purity of the extracted cellulose.

 2) Since cellulose is a soft and fibrous material, PM100 ball mill is not suitable for nano-fibrillation. Hence, a proper explanation can be mentioned in the manuscript. Also, the mean fibre diameter needs to be reported.

 3) Surface area analysis by BET analyzer can be reported to support the increase in surface area due to nanofibrillation.

 4) Innovation in this work is not properly spelled out and is found to be regular work. If any innovation is there should be clearly mentioned.

Is the work clearly and accurately presented and does it cite the current literature?

Partly

If applicable, is the statistical analysis and its interpretation appropriate?

No

Are all the source data underlying the results available to ensure full reproducibility?

No

Is the study design appropriate and is the work technically sound?

Partly

Are the conclusions drawn adequately supported by the results?

Partly

Are sufficient details of methods and analysis provided to allow replication by others?

Partly

Reviewer Expertise:

Agro Machinery Design, Crop Mechanization, Fibre and its Composite, Nano-technology and its application in Textile and Agriculture, Agribusiness Incubation

I confirm that I have read this submission and believe that I have an appropriate level of expertise to confirm that it is of an acceptable scientific standard, however I have significant reservations, as outlined above.

# Reviewer report: Reviewer response for version 1

**DOI:** 10.5256/f1000research.151883.r308132

**Published:** 2024-09-05

**Authors:** Asniza Mustapha

**Affiliations:** 1 Forest Research Institute Malaysia, Kuala Lumpur, Malaysia

Overall, this manuscript is well-written and of good quality. However, I have a few comments on some aspects that may need attention. In general, the manuscript is acceptable.

1. Title: The title is acceptable but a bit too general. It would be more appropriate if the authors could revise it to better reflect the study's focus on the morphological analysis and properties of the obtained CNF.
2. Introduction: Paragraph 2: However, the most common types that are widely used in engineering are CNC and CNF. Please evaluate more on the term “engineering”.
3. Introduction: Paragraph 4: Usually, CNF is extracted from agro-waste materials with the primary aim to turn waste into excellent materials and directly save the environment. I think the term “directly” should be changed to “indirectly”.
4. Introduction: Paragraph 5: Hence, to reduce energy consumption and chemical usage, this work was done to obtain CNF from jute fiber using a chemo mechanical method. How do the authors demonstrate that their method reduces energy consumption and chemical usage? Have any calculations been conducted to support this claim? Please provide an explanation.
5. Table 1: Based on Table 1, it is shown that jute has a low content of lignin, pectin and waxes compared to other types of fibers, which suggests it can easily undergo a bleaching process and have a high fiber strength. How do the authors proved that the fiber exhibited high strength? Please provide an explanation.
6. References: Aslan and Mustafa, 2012. The reference is not included in the references list. Please check.
7. References: Some references are out of guidelines. For example, Kian LK, Saba N, Jawaid M, et al.: A review on processing techniques of bast fibers nanocellulose and its polylactic acid (PLA) nanocomposites. Int. J. Biol. Macromol. 2018. No volume/issue/pages is included.
8. References: Please kindly check the references again, do not include the old references, and check the abbreviation of the journals.

Thank you.

Is the work clearly and accurately presented and does it cite the current literature?

Partly

If applicable, is the statistical analysis and its interpretation appropriate?

Not applicable

Are all the source data underlying the results available to ensure full reproducibility?

Yes

Is the study design appropriate and is the work technically sound?

Yes

Are the conclusions drawn adequately supported by the results?

Yes

Are sufficient details of methods and analysis provided to allow replication by others?

Yes

Reviewer Expertise:

Nanocellulose; Nanocomposites; Biomass; Non-wood based products and processing; Bamboo; Rattan; Palms

I confirm that I have read this submission and believe that I have an appropriate level of expertise to confirm that it is of an acceptable scientific standard, however I have significant reservations, as outlined above.

# Reviewer report: Reviewer response for version 1

**DOI:** 10.5256/f1000research.151883.r274051

**Published:** 2024-06-07

**Authors:** Pawan Kumar Rakesh

**Affiliations:** 1 National Institute of Technology Uttarakhand Srinagar,, Garhwa, Uttarakhand, India

The manuscript focuses on the production of cellulose nanofibers from jute fiber using a chemomechanical approach. The ball milling method was commonly used in the extraction of cellulose nanofiber from natural fibers. It has applications in the cotton and textile manufacturing industries. The manuscript exhibits exceptional writing skills. All the sections have been adequately described. The manuscript is eligible for accepting without any modifications.

Is the work clearly and accurately presented and does it cite the current literature?

Yes

If applicable, is the statistical analysis and its interpretation appropriate?

Not applicable

Are all the source data underlying the results available to ensure full reproducibility?

Yes

Is the study design appropriate and is the work technically sound?

Yes

Are the conclusions drawn adequately supported by the results?

Yes

Are sufficient details of methods and analysis provided to allow replication by others?

Yes

Reviewer Expertise:

I am working on the primary and secondary processing of polymer matrix composites.

I confirm that I have read this submission and believe that I have an appropriate level of expertise to confirm that it is of an acceptable scientific standard.
